# Editorial to the Special Issue “Pathogenesis and Novel Therapeutics in Asthma”

**DOI:** 10.3390/biomedicines11020268

**Published:** 2023-01-19

**Authors:** Stanisława Bazan-Socha, Bogdan Jakieła

**Affiliations:** Department of Internal Medicine, Faculty of Medicine, Jagiellonian University Medical College, 31-008 Krakow, Poland

In recent years, substantial progress has been made in our understanding of asthma pathomechanisms, especially phenotyping. However, asthma is a heterogeneous disease caused by a variety of inflammatory responses. Furthermore, the current pharmacotherapy mainly affects symptoms and does not modify the disease progression, including airway remodeling. Thus, further research on asthma is still of significant importance. In this Special Issue entitled “Pathogenesis and Novel Therapeutics in Asthma”, we addressed various important aspects of asthma, such as disease phenotyping, novel biomarkers, and concepts of customized treatment.

This Special Issue contains seven original articles on the pathomechanism of asthma, including in vitro research investigating novel disease mechanisms and clinical studies on potential disease biomarkers. For example, in an interesting report, Schindler V. et al. [1] used the air−liquid interface culture of bronchial epithelial cells derived from asthma patients and control subjects to study the microRNA profile (miRNA) in released extracellular vesicles (EVs). The authors describe considerable differences in miRNA content, depending on the side of secretion. Over two hundred miRNAs were differentially expressed by comparing EVs isolated from the apical and basolateral cell sides. Interestingly, several miRNAs showed altered expression in cells derived from asthma patients (32 miRNAs in apical EVs, 23 in basolateral) in comparison to the controls. Furthermore, the study evaluated miRNA-associated functions and targets that confirmed differences that were dependent on the site of secretion. For example, apically released EVs contained miRNAs that regulate mammalian targets of rapamycin (mTOR) and mitogen-activated protein kinase (MAPK) signaling pathways, while miRNAs released basolaterally targeted, among others, T and B cell receptor signaling. In conclusion, the study confirms side-specific differences in the miRNA cargo of EVs released by airway epithelium, providing further evidence of the critical role these cells play in the regulation of the immune response in asthmatic airways.

Another original paper investigates the role of NF-κB activation in profibrotic signaling that contributes to airway remodeling in severe asthma. Ramakrishnan R. et al. [2] investigated whether Bcl10 protein, one of the upstream mediators of NF-κB activation, participates in profibrotic signaling in bronchial fibroblasts. The authors demonstrated increased Bcl10 protein expression in airway biopsies and fibroblasts isolated from severe asthma patients. They also confirmed that the selective inhibition of Bcl10 reduced the expression of profibrotic cytokines by activated fibroblasts. This study indicates that targeting Bcl10-associated signaling could be a novel therapeutic option for inhibiting airway inflammation and remodeling in severe asthma.

The study by Bazan-Socha S. et al. [3] documented an increased systemic oxidative stress response in the peripheral blood of asthmatic patients. The authors developed a real-time coumarin boric acid assay (CBA) that enabled a detailed analysis of the kinetics of protein hydroperoxide (HP) formation in serum samples. The study demonstrated increased systemic oxidative stress response in asthma. Furthermore, increased HP formation appeared to be inversely correlated with lung function, as well as being positively associated with inflammatory blood and airway biomarkers. The authors conclude that oxidative stress response is an important component of airway inflammation in asthma, and antioxidant supplementation may benefit asthma management. Interestingly, another study in this Special Issue analyzed whether OmeGo [4], an enzymatically liberated fish oil formulation with potent antioxidant functions, could indeed modify airway inflammation. Currie et al. [4] demonstrated how OmeGo supplementation significantly decreased airway inflammation and remodeling parameters in the murine model of allergic asthma. In many aspects, it showed a similar efficacy as pharmacologic inhibition of the type (T)2-inflammatory response. The data presented by Currie et al. [4] and Bazan-Socha et al. [3] support the need for future clinical studies to evaluate whether a similar approach aimed at reducing oxidative stress could be helpful in asthma.

Special attention must also be placed on the original report published by Ching-Hsiung Lin et al. [5]. These authors assessed the prevalence of fungal sensitization in asthma and checked whether it is associated with specific immune profiles and more severe disease outcomes. Interestingly, they found that about 90% of asthmatics in Taiwan population had fungi-specific IgE in serum, suggesting that this problem may be underestimated worldwide. Furthermore, the serum levels of IL-6 and IL-17A correlated positively with the severity of fungi sensitization; however, only IL-17A was associated with increased asthma-related emergency department visits in the past. Therefore, the Th17-mediated immune response related to fungal sensitization in asthma could be a potential therapeutic target for anti-IL17A therapy. In addition to the possible linkage with fungal allergy, IL-17A has been linked with neutrophilic inflammation in severe asthma [6]. Earlier studies confirmed that this particular inflammatory phenotype is also associated with the increased airway production of leukotriene B4 (LTB4), a lipid mediator acting as a potent chemoattractant for neutrophils. Here, Kwak D.W. et al. [7] used a murine model of steroid-resistant airway inflammation to show that the activation of an LTB4 receptor 2 (BLT2) upregulates granulocyte colony-stimulating factor (G-CSF) synthesis, contributing to the neutrophilic inflammatory response. Interestingly, G-CSF production and subsequent airway neutrophilia depended on the 12-lipoxygenase pathway, which catalyzes the synthesis of the BLT2 ligand, 12(S)-hydroxyeicosatetraenoic acid (HETE). The authors conclude that this pathway may serve as a potential target for treating severe neutrophilic asthma.

Finally, the clinical study by Mormile I. et al. [8] provides exciting data on the factors influential in predicting the response to allergen immunotherapy (AIT). The authors developed a novel rating system called the Predictive Response to Immunotherapy Score (PRIS), which combined both clinical and laboratory measures. This scoring system was next validated in a large cohort of 110 patients who were eligible for sublingual immunotherapy (SLIT) at baseline and at 12 and 24 months follow-up. PRIS was effective in predicting symptom response to SLIT. The parameters that significantly contributed to the scoring included, among others: patients’ age, major disease features (e.g., rhinitis and asthma), and various clinical and laboratory parameters that reflect the severity of allergy (e.g., number of allergen sensitizations, levels of specific IgE). The results of this study suggest that initial PRIS scoring might be recommended as a valuable tool for clinicians to identify patients who may find AIT most beneficial.

Original research studies are followed by a comprehensive review paper by Patel K. and Stokes Peebles, Jr. [9], focusing on the role of PGI_2_ (prostacyclin) in regulating the allergic inflammatory response. PGI_2_ is a metabolic product of the cyclooxygenase pathway, which is constitutively expressed by many airway resident cells and induced during inflammatory conditions. It has been recognized as an effective vasodilator with anti-platelet aggregatory effects since its discovery in 1976 [10]. Furthermore, in vitro and in vivo studies demonstrated its potent anti-inflammatory functions. This review discusses pathways regulating PGI_2_ production and signaling, including various immunomodulatory functions mediated by IP receptors and peroxisome proliferator-activated nuclear receptors. Special attention was paid to cell-specific actions of PGI_2_ and the perspectives of using this pathway in future treatments of allergic diseases and asthma.

This Special Issue ends with a systemic review by Calzetta L. et al. [11], seeking to assess the novel therapeutic agents for asthma therapy investigated in recent Phase I and II randomized controlled trials (RCTs). Based on the literature search and data retrieved from the ClinicalTrial.org database, the authors included 19 clinical trials that had been completed in the last five years (2017–2022). Next, they summarized the types of therapeutic interventions and outcomes, data quality, and potential bias. Overall, the authors identified sixteen classes of novel treatment options for asthma. Among those are anti-inflammatory compounds interfering with the T2 inflammatory response, such as an inhaled form of lipocalin-derived protein inhibiting α subunit of interleukin (IL)-4 receptor (AZD1402), and depemokimab, a long-acting monoclonal antibody (mAb) targeting IL-5. Preliminary RCTs also documented the effectiveness of mAbs against airway alarmins, e.g., those blocking IL-33 (itepekimab and etokimab) or the IL-33 receptor (melrilimab), as well as the mAb fragment against thymic stromal lymphopoietin (TSLP), administered via a dry powder inhaler (ecleralimab). An attractive therapeutic option in asthma, also mentioned by Calzetta et al. [11], could be the blocking of the actions of prostaglandin-D_2_ (PGD_2_), an important lipid mediator released by activated mast cells and eosinophils. Early data suggest the effectiveness of the DP2 receptor antagonist GB001, which was administered orally in improving asthma control, with better effects obtained in eosinophilic asthma.

The authors also discuss the potential treatments that may have wider application in severe asthma, including patients with non-T2 variants of the disease. For example, data on anti-IL-17A mAb (CJM1120) suggest it may improve symptom control in severe non-eosinophilic asthma. Another therapeutic option includes thyrosine kinase inhibitors, such as Bruton’s tyrosine kinase inhibitor remibrutinib, and pan-JAK inhibitor TD-8236. By blocking the signaling associated with tyrosine kinase activity, these drugs have the potential to control overactivated inflammatory pathways in severe asthma. Particular attention should be given to velsecorat, a novel drug acting as a non-steroidal selective glucocorticoid receptor modulator (SGRM). This drug efficiently suppresses inflammation with fewer adverse effects, preferably acting as a transrepressor of steroid receptors. Another therapeutic option for asthma includes the inhalation of sodium channel inhibitor (BI443651), which may have beneficial effects by changing the mucus viscosity in the lower airways. The history of dexpramipexole, an oral drug initially investigated for the treatment of amyotrophic lateral sclerosis, is also interesting. Due to its potent eosinophil-reducing activity, this compound is currently being tested in hypereosinophilic syndrome and asthma. All the mentioned medications seem promising, although larger RCTs are needed to prove their efficiency and safety in asthma and allergy.

In conclusion, this Special Issue represents a novel and interesting view on asthma research. However, further studies elucidating the complex mechanisms of asthma (particularly non-T2) will be of great importance. Furthermore, the newly proposed asthma therapies require validation in different phenotypes and endotypes of the disease.
